# Temperature‐dependent responses to light and nutrients in phytoplankton

**DOI:** 10.1002/ecy.70027

**Published:** 2025-03-03

**Authors:** Anna Lena Heinrichs, Anika Happe, Apostolos‐Manuel Koussoroplis, Helmut Hillebrand, Julian Merder, Maren Striebel

**Affiliations:** ^1^ Institute for Chemistry and Biology of the Marine Environment (ICBM), Carl‐von‐Ossietzky University of Oldenburg, School of Mathematics and Science Oldenburg Germany; ^2^ Laboratoire Microorganismes Genome et Environnement (LMGE) UMR CNRS 6023, Université Clermont Auvergne Aubière Cedex France; ^3^ Helmholtz Institute for Functional Marine Biodiversity (HIFMB), Carl‐von‐Ossietzky University of Oldenburg Oldenburg Germany; ^4^ Alfred Wegener Institute, Helmholtz‐Centre for Polar and Marine Research [AWI] Bremerhaven Germany; ^5^ Department of Global Ecology Carnegie Institution for Science Stanford California USA; ^6^ Present address: Instituto Gulbenkian de Ciênca (IGC) Oeiras Portugal

**Keywords:** gradient design, growth, interactive‐effects, light:nutrient ratio, resource limitation, temperature dependence

## Abstract

Nutrients and light are major resources controlling growth, biomass, and community structure of phytoplankton. When looking at those resources individually, resource uptake and biochemical transformation, and thereby also the demand for resources, have been shown to be temperature‐dependent. However, there is still a lack of understanding of how temperature controls the response to multiple resources, although simultaneous limitation by multiple resources is common for single species and whole communities. We conducted a multifactorial, gradient‐design experiment growing four freshwater phytoplankton species under 125 combinations of temperature, light, and nutrients (5 × 5 × 5 levels). In three of four species, we found evidence for an interactive effect of light and nutrients on growth that was modulated by temperature. The effect of high‐level supply of both resources on algal growth rate generally exceeded the sum of their individual effects. Conversely, the lowest growth rates occurred not necessarily at the lowest level of both resources but at the most extreme light:nutrient supply ratios (either only light or nutrients were at highest supply level but the other resource remained at low supply). These interactive light‐nutrient effects were modulated by temperature, resulting in highest growth rates when both resources and temperature were highest. Our study demonstrates that temperature modulates the magnitude of the interactive light‐nutrient effect on phytoplankton growth. Consequently, these findings highlight the importance of considering temperature to understand the limitation by multiple resources and show that growth responses would be over‐ or underestimated when these interactions are not taken into account. Our results provide a first indication that the resource‐dependent growth of phytoplankton will change in a warming world when considering multiple resources.

## INTRODUCTION

Limiting resources constrain the ecological niche of animals and plants, as they lower nutrient uptake and growth rates (Tilman et al., 1982). These functional and numerical responses to resource availability are classically studied in response to a single limiting factor as proposed by Liebig's Law of the Minimum (Sprengel, 1827; von Liebig, 1840). In this view, only the most limiting resource with the least supply compared with the demand determines the maximum biomass yield of a population. However, accumulating evidence shows that in natural communities, organisms often are simultaneously limited by multiple resources (Gleeson & Tilman, 1992). The limitation by multiple resources is clearly the rule rather than the exception at community scales (Allgeier et al., 2011; Elser et al., 2007; Harpole et al., 2011), caused by different resource demands between genotypes and species (Arrigo, 2005). Additionally, there is also the possibility of biochemical limitation by multiple resources at the individual species level (Arrigo, 2005; Danger et al., 2008; Sperfeld et al., 2016). In this case, the presence of one resource is needed to take up or transform another resource. Autotrophic organisms are particularly susceptible to such biochemical co‐limitation since their uptake mechanisms for resources are often interdependent (Pahlow & Oschlies, 2009).

The empirical assessment of multiple‐resource limitations relies mainly on nutrient addition experiments (Elser et al., 2007). In both terrestrial and aquatic primary producer communities, the magnitude of an organism's response (e.g., biomass increase) to multiple nutrients may range from sub‐additive to super‐additive, the latter indicating either simultaneous or independent co‐limitation (Harpole et al., 2011). In addition to multiple‐nutrient limitation, in aquatic environments, special attention has been given to nutrient–light limitation that influences photoautotrophic organisms at biochemical (Eppley et al., 1970) stoichiometric (Sterner et al., 1997), biomass and production levels (Carey et al., 2007; Dubourg et al., 2015). In aquatic systems, the gradients of nutrient concentration and light intensity differ along the water column, so the condition for optimal growth, that is, a balanced ratio of resources (Sterner et al., 1997), is not necessarily given: Irradiance decreases exponentially with water depth, whereas nutrient availability often increases toward deeper water. These inverse resource gradients with water depth lead to spatially structured resource limitations, and competition outcomes for light and nutrients deviate from the prediction made for well‐mixed surroundings (Ryabov, 2012). Light and nutrients are both essential resources for photoautotrophs, and the processes of nutrient uptake and photosynthesis that determine the organism's growth are interdependent; carbon fixation by photosynthesis is limited by nitrogen (N), as this is needed for the synthesis of pigments (Pahlow, 2005; Pahlow & Oschlies, 2009). Consequently, increased light can only increase primary production if nutrient demands are covered, whereas nutrient limitation reduces the potential investment in pigment synthesis, which would lead to a suboptimal light harvesting (Mette et al., 2011). Conversely, N‐uptake and transformation can be limited by light, as the light‐dependent part of photosynthesis provides ATP and NADPH for anabolic processes (Falkowski & Stone, 1975)25

It can further be assumed that the resource range where a limitation by multiple resources occurs changes along other environmental gradients. Temperature is one of the main environmental drivers that imposes fundamental constraints on the metabolism of organisms and influences phytoplankton metabolic traits such as the half‐saturation constant (Qu et al., 2018), nutrient uptake rates (Gao et al., 2000), and minimum resource requirements (Lewington‐Pearce et al., 2019). Consequently, there is abundant evidence of the interactive effect of temperature and single resources (either nutrients or light) on phytoplankton growth. The availability of nutrients shapes the response to temperature, whereas nutrient limitation has been shown to reduce the temperature sensitivity which results in a flattening of the temperature response curve (Aranguren‐Gassis et al., 2019; Maranon et al., 2018). As a consequence of the temperature‐nutrient effect, low nutrient concentrations reduce the temperature optimum for growth (Bestion et al., 2018; Thomas et al., 2017) and inhibit community biomass production during a heat wave (Hayashida et al., 2020). The temperature optimum (*T*_opt_) in turn correlates with the irradiance optimum (*I*_opt_), so species that have their temperature optimum at higher temperatures prefer higher light intensities (Bouterfas et al., 2002; Edwards et al., 2016). In addition to the temperature‐light or temperature‐nutrient interaction effect on growth, temperature influences cellular N:P stoichiometry, indicating that temperature also controls the resource space where co‐limitation can occur as higher temperatures increase ribosomal efficiency and thus reduce P‐demand, shifting optimal ratios and thus the most likely co‐limitation region to higher N:P (Thrane et al., 2017; Toseland et al., 2013). Extending the temperature dependence on resource use by including light, Arteaga et al. (2014) showed global ocean patterns of different resource limitations between light, N, and P that changed along latitudinal gradients and seasons, suggesting that resource limitations are temperature‐dependent for phytoplankton elemental composition.

Despite the amount of evidence for a temperature‐dependent light or nutrient effect on phytoplankton growth, there is still a lack of knowledge of how temperature modulates the response to multiple resources (i.e., light and nutrients) in phytoplankton growth. To understand the type of interaction between temperature, light, and nutrient supply, and thus what role temperature plays in the light‐nutrient effect, we manipulated the availability of light and nutrients along a temperature gradient and monitored the responses of four freshwater phytoplankton species. In a controlled laboratory experiment, we grew four monoclonal species at 5 × 5 × 5 combinations of temperatures, nutrient concentrations and light intensities, and measured their maximum growth rates. We hypothesized that light and nutrients interactively affect phytoplankton growth rather than influencing the growth independently (H1) and that the interactive effect of light and nutrients depends on temperature (H2). To test H1 and H2, we fitted generalized additive models (GAMs), with and without such interaction terms, and compared their performance using likelihood ratio tests and the corrected Akaike information criterion (AIC_c_): A significant likelihood ratio test and lower AIC_c_ in a model with resource interaction term, relative to the model without interaction term, would support H1. H2 would be supported if including the temperature‐resource interaction enhances the model's performance even more, providing empirical evidence that the dependency on both resources, light and nutrients, changes with temperature.

## METHODS

We conducted a laboratory experiment using four freshwater phytoplankton species, *Scenedesmus armatus*, *Coelastrum astroideum*, *Staurastrum manfeldtii*, and *Cosmarium botrytis*, isolated from the lake Grafschaftssee (Germany, 53°33′005″ N; 7°58′049″ E) in July 2020. We selected these species to cover a range of cell sizes (39–16,900 μm^3^) and growth characteristics as well as relative abundance in the natural community (species hereinafter referred to as *Scenedesmus*, *Coelastrum*, *Staurastrum*, and *Cosmarium*). We reduced the trait variability within the population by using monoclonal monocultures. Thereby, we focused on biochemical co‐limitation (multiple resources needed by a single cell) and excluded co‐limitation due to different resource demands based on genetic variation (different genotypes in the population). Species isolation was conducted using a micropipette (Andersen & Kawachi, 2005) under an inverted microscope (Leica, Germany). We repeated the isolation steps until a monoclonal culture was obtained for each species (cultures were unialgal but not axenic). Prior to the start of the experiment, species were cultivated for 6 weeks in 1/4 WC Medium (Guillard & Lorenzen, 1972) at 18°C and a light intensity of 70‐μmol photons m^−2^ s^−1^ with a 12/12 light/dark regime.

### Experimental design

We performed a multiple‐gradient experiment (Table 1) applying five levels of temperatures (10–30°C), five light intensities (36–264‐μmol photons m^2^ s^−1^), and five nutrient concentrations (N and P with a constant ratio) for each species, resulting in a total of 500 experimental units. The experiment was conducted in cell culture flasks (50 mL, Sarstedt AG & Co. KG) using a total volume of 40 mL. The bottles were incubated in the indoor mesocosms at the ICBM Wilhelmshaven (Gall et al., 2017) to ensure full light spectrum and temperature control. To obtain five different temperature levels, all samples were incubated using floating plastic boxes on the water surface of the mesocosm providing the respective temperatures, which we controlled via data logger (Hobo Pendant, Onset, Bourne, MA, USA) exposed in the boxes (Appendix S1: Figure S1). For the light treatments (Table 1), we used two light‐emitting diode (LED) modules per mesocosm (Evergrow IT2040; Shenzhen Sanxinbao Semiconductor Lightning Co. Ltd) positioned above each mesocosm and adjusted light intensity by covering the floating plastic boxes with four different neutral gray filter foils (LEE Filter Nos. 209, 210, 211, and 298), which reduce intensity but retain the full spectrum (Hintz et al., 2021). The light reduction (in percentage) by the light filter foils was measured with a spherical PAR sensor (US‐SQS/L Submersible Spherical Mirco Quantum Sensor, Walz, with LI‐250A, LI‐COR) that was covered by the respective light filter foil and placed below a LED panel. The full light intensity (100%) in the mesocosm without light filter foil was measured at the surface of the water (position of the plastic boxes) in the mesocosms and the absolute light intensity for the other light levels was calculated with the full light intensity and the amount of light reduction (in percentage) induced by the respective filter foil. For the nutrient gradient, we added nitrogen (N, as NaNO_3_) and phosphorus (P as K_2_HPO_4_) in different concentrations but same ratio at the beginning of the experiment as a single addition (Table 1). A bioassay conducted with the initial community where the species originated from showed a co‐limitation of both N and P (Appendix S1: Figure S2). To avoid limitations by other elements, we added nutrients, except N and P, according to 1/4 WC growth medium (Guillard & Lorenzen, 1972). Note: The targeted N and P additions differed from the actual nutrient additions shown in Table 1, so the actual N and P ratio deviated from the planned 16:1 ratio (mean molar N:P ratio = 16.2, SD = 2.3).

**TABLE 1:** The experimental treatments (temperature [in degrees Celsius], light intensity [in micromoles of photons per square meter per second], and nutrients [in micromoles per liter]) were set up in a combined gradient design (5 × 5 × 5) resulting in 125 treatments per species whereby nitrogen (N) and phosphorus (P) were added together as the nutrient treatment.

Temperature	Light	Nutrients
		N/P
10	36	1.8/0.1
15	62	13.2/0.9
20	135	26.3/1.7
25	183	34.3/2.2
30	264	46.5/3.0

### Sampling

We measured the optical density (OD, absorbance at 440 nm) and the raw fluorescence (RFU, excitation = 395 nm; emission = 680 nm) using a microplate reader (Synergy H1, BioTek instruments) to track the biomass development over time. Flasks were gently shaken before 0.5mL subsamples were removed for sampling under sterile conditions (clean bench, Berner) and measured using 48‐well microplates (SARSTEDT AG & Co. KG) every other day. After sampling, we placed the cell flasks randomly in their respective light treatment boxes in the incubators. Final samples were taken when the saturation phase (carrying capacity) was reached. We defined the saturation phase to be reached as soon as the OD did not increase for at least six following days (three samplings). Hence, we took final samples at different times of the experiment for different treatments, depending on the time they reached the saturation phase (Appendix S1: Figure S3).

### Growth rates

For growth rates determination *r* (day^−1^), we used the R package “*growthrates*” to capture the maximum slope of the growth curve, using the RFU measurements. The used function “*fit_easylinear*” (Hall et al., 2014) relies on the slope estimates from a linear trend encompassing at least four data points. Since *Staurastrum* and *Cosmarium* are desmids and can produce some mucous that surrounds the cells as gelatinous layer (mucous influences the OD but not the RFU), the OD and RFU data showed in some treatments opposite trends over time. Therefore, we decided to use the RFU data for all species to determine the growth rates. The RFU based data provided growth curves that were independent of bacteria and mucous production and showed less variability (see Appendix S1: Figure S4 for incubation curves based on OD data, Appendix S1: Figure S5 for comparison between growth estimates on RFU and OD basis, and Appendix S1: Table S1 for outcomes of statistical analyses with growth rates based on OD data).

### Statistical analyses

We performed the complete statistical analysis in R (version 3.6.2, the R Foundation for Statistical Computing Platform). Sample sizes for the species *Cosmarium* and *Staurastrum* were reduced for further analyses due to contaminations of *Coelastrum* (see Table 2 for sample size).

**TABLE 2:** Model comparison based on corrected Akaike information criterion (AIC_c_) for all species (see for model validation plots, and for growth rate predictions by the different models Appendix S1: Figures S6 and S10).

Model	Coelastrum	Cosmarium	Scenedesmus	Staurastrum
	AIC_c_	AIC_c_	AIC_c_	AIC_c_
Independent effects (null model)	−225	−248	−280	−204
Interactive resource effect (H1)	−Δ22	−Δ6	−Δ9	+Δ2
Interactive temp‐resource effect (H2)	−Δ26	−Δ17	−Δ33	+Δ6
No. Obs.	125	109	124	100

#### Interactive resource effect on growth (H1)

To provide evidence for an interactive light‐nutrient effect on species‐specific growth rates (H1), we used two GAMs for each species, respectively: The first model reflects independent effects of temperature, light, and nutrients (hereafter *null model*) (Equation 1). The second model includes an interaction term of light and nutrients (hereafter *Resource Interaction model*) on species‐specific growth rates (Equation 2). More specifically, for the *null model*, temperature, light, and nutrients were modeled as smooth functions, while for the *Resource Interaction model*, we added a linear pairwise two‐way interaction between light and nutrients (Equation 1). We compared the models via corrected AIC (AIC_c_) and likelihood ratio test (Stasinopoulos & Rigby, 2008) using the R package “*MuMIn*” (Bartoń, 2023). While the likelihood ratio test assesses whether including the interaction term significantly enhances the model's goodness of fit (Lewis et al., 2011), the AIC balances the model's goodness of fit with its complexity, aiming to find a parsimonious model that avoids overfitting, thus identifying the most appropriate model (among our hypothesis‐based model candidates) in line with the data. As such, we are finding strong support for the interactive effect between nutrients and light when the Resource Interaction model has a distinctly lower AIC_c_ than the null model (ΔAIC_c_ > 2) and the likelihood ratio test rejects the null model (*p* < 0.05). For model fitting we used, the R package “*gamlss*” (Stasinopoulos & Rigby, 2008), and for model validation the R package “*gamlss:ggplots*” (Stasinopoulos et al., 2022).

#### Temperature effects on the interactive resource effect (H2)

In order to test whether temperature influences the interdependent response to both resources (i.e., interactive resource effect) (H2), we fitted a third GAM that includes all second‐ and third‐order temperature‐resource interaction terms (Equation 3) (hereafter *temperature‐resource model*). A lower AIC_c_ for the temperature‐resource model and a significant likelihood ratio test compared with the resource interaction model indicate that growth rates are better predicted by a model that considers the interactive temperature‐resource effects, thus supporting H2.

Null model (H0):

(1) $$E\left(r\right)=a+f_{1}\left(T\right)+f_{2}\left(N\right)+f_{3}\left(L\right)$$

Resource Interaction model (H1):

(2) $$E\left(r\right)=a+f_{1}\left(T\right)+f_{2}\left(N\right)+f_{3}\left(L\right)+b_{1}\mathrm{NL}+b_{2}\mathrm{TL}+b_{3}\mathrm{TN}$$

Temperature–Resource Interaction model (H2):

(3) $$E\left(r\right)=a+f_{1}\left(T\right)+f_{2}\left(N\right)+f_{3}\left(L\right)+b_{1}\mathrm{NL}+b_{2}\mathrm{TL}+b_{3}\mathrm{TN}+b_{4}\mathrm{TNL}$$

The models were created for each species separately. $E\left(r\right)$ is the expected growth rate r; $f_{1},f_{2},f_{3}$ are smooth functions of the explanatory variables temperature (T), nutrients (N), and light (L); $b_{1},b_{2},b_{3}$ the coefficients of linear two‐way interactions between explanatory variables; $b_{4}$ the coefficients of the three‐way interaction; and a the model intercept. The smooth functions allow for nonlinear effects on growth of the used factors. Although we are aware that the nutrient–light interaction might also be nonlinear, we only considered a linear interaction term here for simplicity, ensuring the interaction is monotonic and not generating an overly complex model in respect to the size of the data. The model assumes normally distributed (Gaussian) errors (see Appendix S1: Figure S6 for model validation plots for the temperature‐resource model, and Appendix S1: Figures S7–S10 for model validation of all three models [null model, H1, H2]).

The growth rate predictions by the model with the best AIC_c_ were used for data visualization in form of response surface plots. For the visualization of the observed growth rates as well as predictions by the other models along the resource gradients and temperatures see Appendix S1: Figure S10.

**FIGURE 1:**
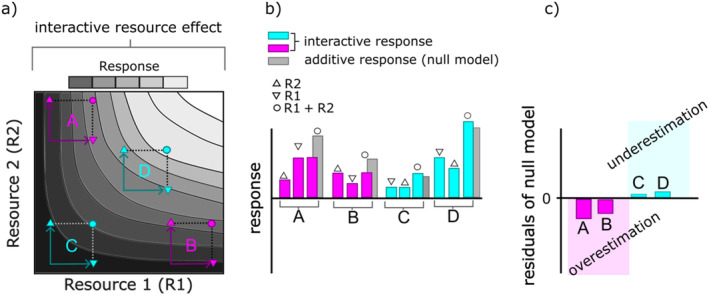
Conceptual figure of possible responses to gradients of two resources. (a) On a response surface, different responses to multiple resources can be found resulting in numerous limitation scenarios depending on the supply ratio and concentration of the resources (R1, R2) (Sperfeld et al., 2016). Black solid lines are resource‐dependent growth isoclines that indicate equal growth at changing resource availabilities. (b) The interactive effect of the two resources can deviate from the additive response (no interaction, gray bar) resulting in higher or lower responses. (c) To classify at which resource conditions along the resource gradients the interactive effect would be under‐, or overestimated by an additive null model, the residuals of the additive model can be used. Positive residuals mean that the observed response is higher than the additive response and thus would be underestimated by the null model (turquoise bars). Negative residuals mean that the observed response is lower than the additive response and thus would be overestimated by the null model (purple bars).

For the species where the temperature‐resource model had the best AIC_c_, we compared the observed growth rates with those that were predicted by the null model using normalized quantile residuals (standardized difference between observed growth rates and predicted growth rates) (Dunn & Smyth, 1996). We visualized the residuals along the predicted values as well as the used light:nutrient ratios, for each temperature separately, which allowed us to identify at which resource and temperature conditions the growth rates are under‐, or overestimated by the null model (*Staurastrum* was excluded as the growth rates were better predicted by the null model, rejecting H1 and consequently H2). In general, systematic deviations of the residuals from 0 indicate a misspecified model, where systematically positive residuals present higher observed growth rates and thus underestimated growth rates by the null model, and systematic negative values present lower observed growth rates and thus overestimated growth rates by the null model (Figure 1). If these residual patterns vanish in models incorporating interaction terms provides additional support for such interactions modulating phytoplankton growth rates.

## RESULTS

### Interactive resource effect on growth (H1)

**FIGURE 2:**
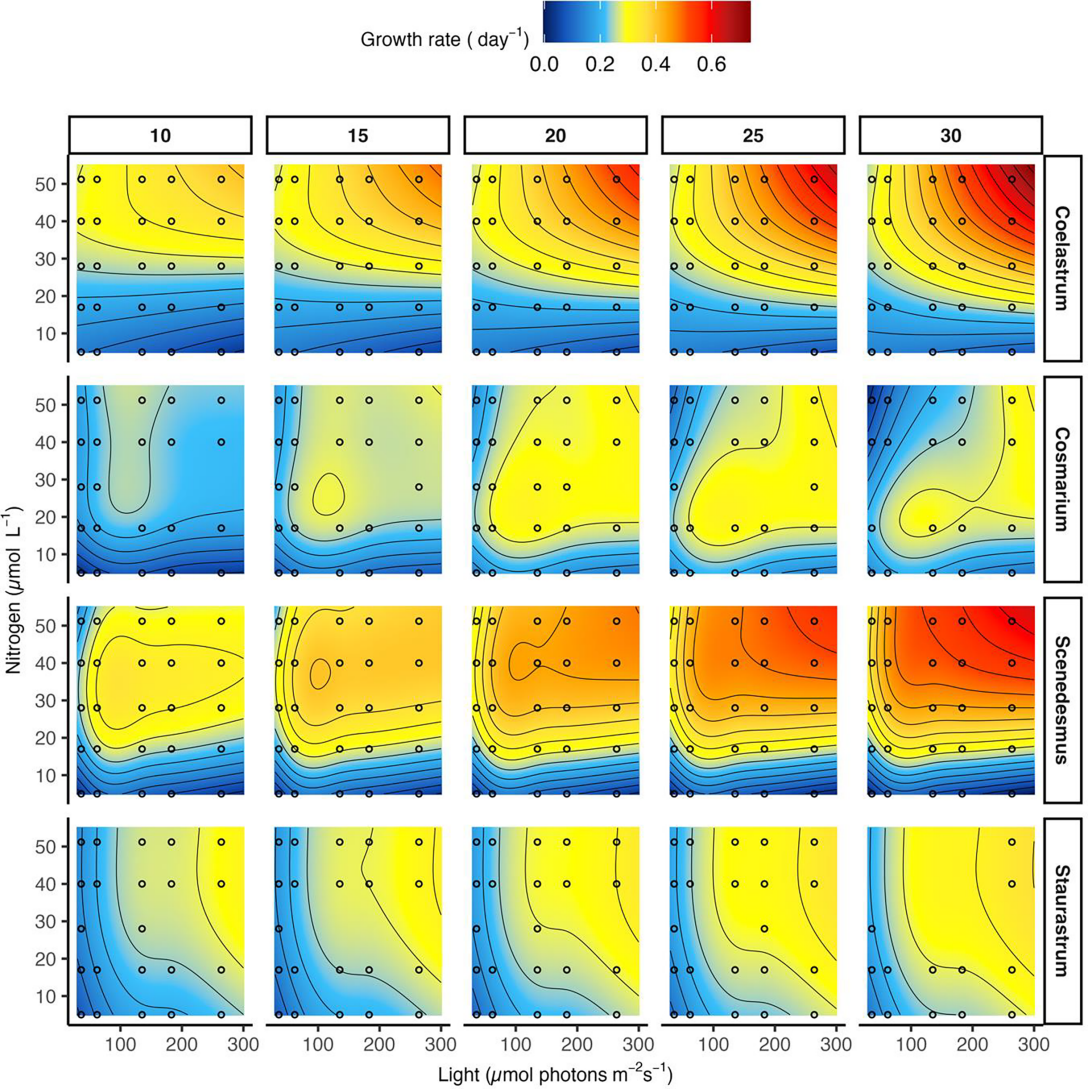
Response surface for species‐specific growth rates (day^−1^), predicted by the best generalized additive model (determined with corrected Akaike information criterion and *Likelihood Ratio Test*), along resource gradients and across temperatures (10–30°C). Except for *Staurastrum*, the model that fitted the observed growth rates best was the temperature‐resource model. For *Staurastrum*, the null model was used for visualization. Dots represent the resource concentrations used in the experiment. Black lines are growth isoclines and were created with *geom_contour* in ggplot. Nitrogen concentration was used as a representative parameter for nutrient addition as the molar ratio between nitrogen and phosphorus was kept constant (see Table 1 for corresponding phosphorus concentration). Response surface plots that contain only the observed growth rates can be found in Appendix S1: Figure S10.

For three of the four species, observed growth rates were better predicted by the resource interaction model that included the interaction term between light and nutrients than by the model without interactions (null model), including only the independent effects of temperature and both resources (based on the AIC_c_ difference to null model, ΔAIC_c_, and a likelihood ratio test *p* < 0.05) (Table 2). One exception was *Staurastrum*, whose growth rates were better predicted by the null model than by the resource interaction model due a higher AIC_c_ for the resource interaction model and a nonsignificant likelihood ratio test (*p* > 0.05) (Table 2 and Appendix S1: Figures S7–S9). Therefore, light and nutrients interactively affected the growth rates in three of the four species resulting in highest growth rates when both resources together were at highest level and lowest when only one resource was enhanced but the other was kept at the lowest level (hereafter *extreme resource supply ratio*) (Figure 2).

### Interactive temperature–resource effect (H2)

To test whether the observed interactive resource effect is temperature‐dependent, we compared the resource interaction model with the temperature‐resource interaction model. For three of the four species (except for *Staurastrum*), the temperature‐resource model showed distinct lower AIC_c_ values than the resource interaction model (Table 2 and Appendix S1: Figures S7–S9), and the likelihood ratio test rejected the resource interaction model (*p* < 0.05). Therefore, temperature did not only increase predicted growth rates gradually but influenced the interactive effect of light and nutrients on species‐specific growth rates. This interactive temperature‐resource effect resulted in highest growth rates when all three factors were at highest level (Figure 2).

**FIGURE 3:**
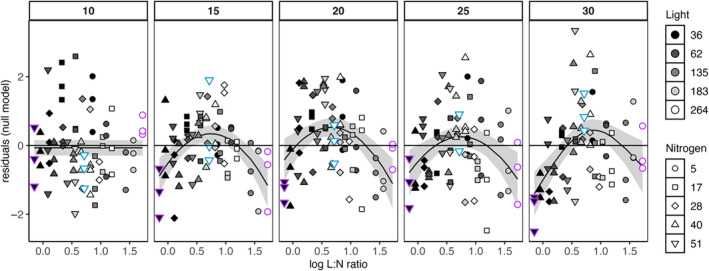
Normalized quantile residuals of the null model (i.e., standardized differences between the observed growth rates and those predicted by the null model) along the supplied light:nutrient ratios (L:N) across temperatures (panel grids). Plot includes the data of the species *Coelastrum*, *Cosmarium*, and *Scenedesmus* (*Staurastrum* was excluded as this species does not show a temperature‐resource effect). Smooth solid lines and CIs were created via generalized additive model in ggplot and indicate a systematic deviation from zero. Shapes present the nutrient concentrations in nitrogen (but see Table 1 for phosphorus concentrations) and filled colors the light intensities. Colored contours (turquoise and purple) present the resource treatments at highest and intermediate (turquoise) resource supply ratio, and extreme (purple) resource supply.

The visualization of the normalized quantile residuals of the null model along the light:nutrient ratios for each temperature showed clearly identifiable residual patterns (Figure 3) (while the *temperature‐resource model* exhibits no significant residual patterns; see Appendix S1: Figures S7 and S8 for residual patterns of all models). At extreme light:nutrient supply ratios (Figure 3, purple shapes) the *null model* overestimated the growth rates with lower and more negative values the higher the temperature (Figure 3). At the same time, the null model underestimated the growth rates at intermediate light:nutrient supply ratios, especially when both resources were at highest level (Figure 3, turquoise triangles), with a more severe residual pattern the higher the temperature (Figure 3). Therefore, the overestimation of growth rates under extreme resource supply ratios and the underestimation of growth rates at intermediate resource supply ratios, particularly when both resources are high, increased with rising temperature, if temperature‐resource interactions were not accounted for.

## DISCUSSION

The objective of this study was to test whether the temperature dependence of metabolism also affects the response to multiple resources. The multifactorial gradient design used here allowed us to show the interdependent effect of light and nutrients on phytoplankton growth and to provide experimental evidence for the temperature dependence of multiple‐resource limitations within phytoplankton species. To our knowledge, our study is the first that systematically assesses and quantifies the combined effect of temperature and multiple resources, light and nutrients, on phytoplankton growth.

The interactive resource effect we found confirms H1 and is not surprising as the photosynthetic apparatus is strongly coupled with the availability of nitrogen and therefore reflects the mechanistic links between carbon fixation and chlorophyll synthesis. Consequently, nutrient limitation reduces the potential investment in pigment synthesis, which can lead to a suboptimal light use efficiency (Mette et al., 2011). While chlorophyll content and thus the nitrogen demand for photosynthetic pigments generally decreases with increasing light intensity (Eppley & Sloan, 1966), increased light can only increase photosynthesis and thus growth (Eppley & Sloan, 1966) if nutrient demands are covered which is in line with our findings. Accounting for temperature‐dependent interactive resource effects (H2) leads to significantly improved models without overfitting (see Appendix S1: Figures S7 and S8), confirming H2 and thus showing that the interdependent response to light and nutrients is modulated by temperature. The one exception was *Staurastrum*, which showed the best growth rate predictions by the null model, rejecting H1 and H2. However, it should be noted that especially at highest temperature (30°C) and high‐resource conditions *Staurastrum* showed a number of missing values due to contaminations which could have influenced the model outcome (see Appendix S1: Figure S10 for missing values). Moreover, the growth rates of *Staurastrum* based on OD data showed a better model outcome when accounting for the resource interaction (H1) compared with the model without resource interaction (null model), strengthening the overall results of this study (see Appendix S1: Table S1 for model comparison based on OD data). A further potential caveat when interpreting the results of this study is the fact that there was no acclimation of the cultures prior to the experiment. Organisms need time to respond to their environment to optimize performance (i.e., gradual acclimation; Fey et al., 2021) by for instance maximizing light absorption and adjusting nutrient uptake rates (Cáceres et al., 2019; Lewis et al., 2019). Thus, it is likely that the used species might have underperformed during the first days of the experiment relative to acclimated populations. However, we consider this bias to be minor as we ran the experiment until the populations reached their stationary phase (minimum of 12 days) and estimated the maximum growth rate in this period of time.

Based on the evidence from the superior H2 model indicating temperature‐dependent interactive resource effects, we show that phytoplankton growth is promoted under high‐resource supply of both light and nutrients at warm temperatures rather than at lower temperatures. This positive interactive effect resulted in maximum growth rates at highest levels of temperature, light, and nutrients. Although we did not find other studies that tested for the combined effect of all three factors together, there are multiple studies focusing either on temperature‐light or temperature‐nutrient effects on phytoplankton. These two‐way interactions support the promoting temperature effect at high‐resource concentrations we found and suggest a stronger dependence on resources with rising temperature. For instance, along an increasing light gradient, studies reported higher maximum population growth rates (at *I*_opt_) at warmer than colder conditions (when the temperature is below *T*_opt_) (Boumnich et al., 1990; Bouterfas et al., 2002; Hammer et al., 2002; Spilling et al., 2015). Further, Hayashida et al. (2020) found that rising temperatures due to marine heat waves yielded stronger algal blooms in nutrient‐rich than nutrient‐poor waters. Dai et al. (2023) showed that warming favors coastal phytoplankton blooms with an effect size that was positively influenced by nutrient enrichment.

Previous studies showed that the required light intensity as well as the nutrient concentration at which growth is maximized increases as temperature rises but also the growth rates itself (Baker et al., 2016; Qu et al., 2018; Thomas et al., 2017). This means that on one hand a higher supply is required to achieve maximized growth rates as temperature rises, but on the other hand, when the supply is met, higher rates can be achieved. In support of this, other studies showed that the higher demand for nutrients with rising temperatures (Lewington‐Pearce et al., 2019; Qu et al., 2018) makes phytoplankton living in nutrient‐poor waters more vulnerable to high temperatures (Aranguren‐Gassis et al., 2019). Aligning with these findings, a freshwater mesocosm experiment showed a positive biomass response to warming at high nutrient supply, but negative at nutrient‐limiting conditions (Verbeek et al., 2018). Our results perfectly fit into these findings. While in our study rising temperature promoted the positive effect of multiple resources at intermediate supply, growth rates remained constantly low across temperatures at extreme light:nutrient supply ratios. These findings coincide with previous studies showing a weaker response in growth along a temperature gradient when nutrients were limiting (Aranguren‐Gassis & Litchman, 2020; Maranon et al., 2018). In our study, the null model increasingly overestimated the growth rates at extreme resource supply ratios the higher the temperature suggesting that warming narrows the required nutrient:light ratios to promote growth. Klausmeier et al. (2004) manipulated phytoplankton growth rates using a chemostat setup and showed that the flexibility of the cellular N:P ratio is reduced at high growth rates but is more dependent on the supplied nutrient ratios at low growth rates. The limited tolerance of the cellular N:P ratio indicates that fast‐growing phytoplankton requirements are more stoichiometrically constrained than slow‐growing phytoplankton (Hillebrand et al., 2013; Klausmeier et al., 2004). Although these studies focused on the ratio between two nutrients (N:P), the need for a certain resource ratio at high growth rates (and thus high temperatures) may also underlie our results for different light:nutrient supply ratios.

In conclusion, our results on the temperature‐dependent response to multiple resources agree with studies that tested for temperature‐dependent responses to single resources. Additionally, we showed that positive light effects on phytoplankton growth rates are highest in warm and nutrient‐rich conditions, nutrient effects are highest in warm high‐irradiance conditions, and temperature effects are highest at high‐resource supply in intermediate ratios. We can conclude that temperature modulates the limitation by multiple resources in predictable ways, which opens the opportunity to improve parametric models trying to predict global change responses in aquatic systems.

## IMPLICATIONS

Anthropogenic global change alters surface temperatures in aquatic ecosystems (Pachauri et al., 2014), nutrient availability, and light conditions. Understanding the mechanisms that shape phytoplankton responses to temperature‐resource interactions is therefore crucial for predicting how climate change and human impact will alter phytoplankton productivity at the basis of aquatic food webs. Ignoring these interactions would overestimate or underestimate the impact of multiple resources under different temperature conditions that shape resource competition and community structure.

## AUTHOR CONTRIBUTIONS

Anna Lena Heinrichs and Maren Striebel designed the study. Anna Lena Heinrichs and Anika Happe performed the experiment. Anna Lena Heinrichs wrote the first draft of the manuscript and conducted the initial analysis with substantial input from Apostolos‐Manuel Koussoroplis, Julian Merder, Helmut Hillebrand, and Maren Striebel. All authors contributed critically to the drafts and gave final approval for publication.

## CONFLICT OF INTEREST STATEMENT

The authors declare no conflicts of interest.

## Supporting information


Appendix S1.

## Data Availability

Data and code (Heinrichs et al., 2024) are available in Zenodo at https://doi.org/10.5281/zenodo.14334970.
