# Supplementary material for: Temperature‐dependent responses to light and nutrients in phytoplankton
Source: Ecology. 2025 Mar 3;106(3):e70027. doi: 10.1002/ecy.70027 (PMC11874050; doi:10.1002/ecy.70027)
Supplement: Supplementary file 1 — Appendix S1. [file ECY-106-e70027-s001.pdf]

Appendix S1

Supporting Information: Anna Lena Heinrichs, Anika Happe, Apostolos-Manuel Koussoroplis, Helmut Hillebrand, Julian Merder and Maren Striebel. Temperature-dependent responses to light and nutrients in phytoplankton. Ecology.

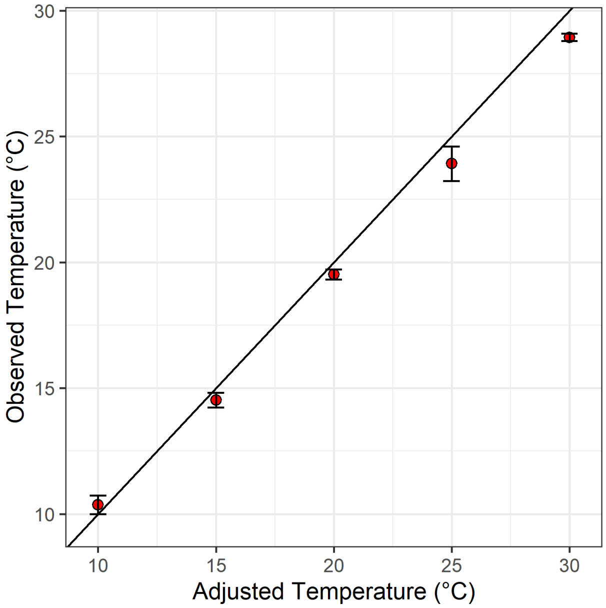

Figure S1 Documented mean temperatures for each temperature level during the experiment. Measured every 20 minutes with continuous data logger (Hobo Pendant ®, Onset, Bourne, MA, USA). Adjusted temperatures represent the targeted temperature level. Observed temperatures differed slightly from the adjusted temperatures for the experiment.

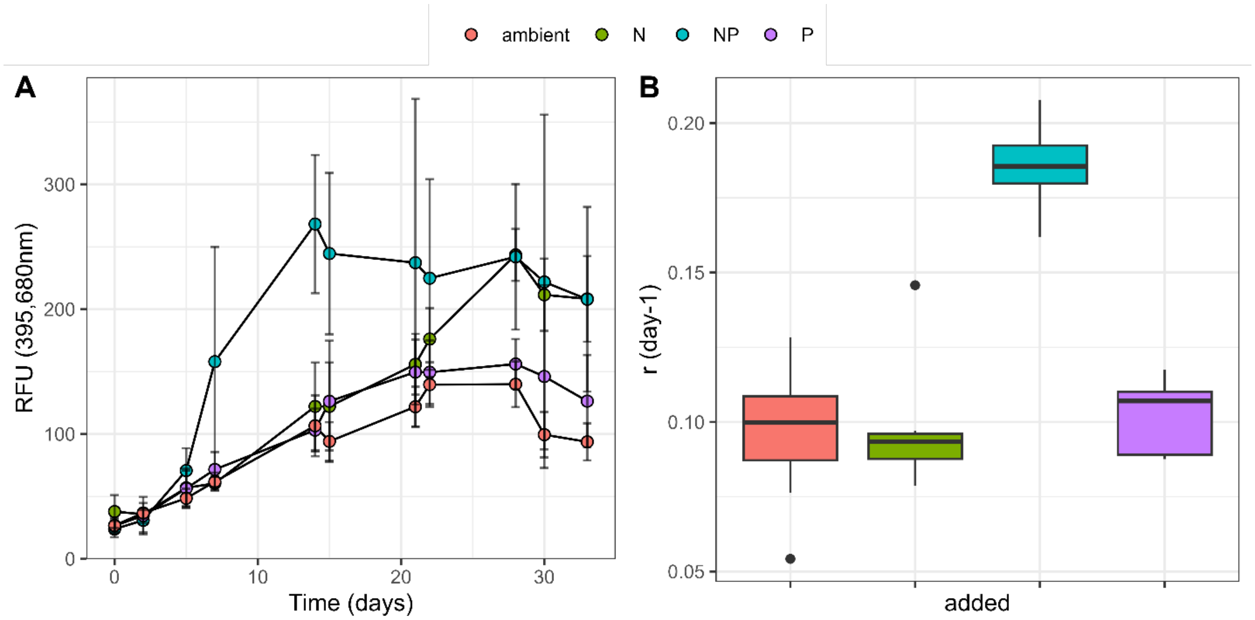

Figure S2 Bioassay of the natural lake community before isolation of species occurred. Simultaneously with the start of species isolation, a bioassay was performed in order to determine the limiting nutrient(s) for the natural community. The preliminary experiment was conducted in 24-well microplates (SARSTEDT AG & Co. KG) with a volume of 2 ml and consisted of four treatments with eight replicates each: An ambient treatment without nutrient addition, an addition of phosphate (KH_2PO_4), an addition of nitrogen ($NaNO_3$) and an addition of both nutrients. Nutrient concentrations were added based on WC Medium. During the bioassay experiment, the microplates were stored in a climate chamber under fixed environmental conditions (18 °C, 70 µmol photons m^{-2} s^{-1}, 12:12 light:dark period). The optical density was measured using a microplate reader (Synergy H1, BioTek instruments) every other day. N and P concentrations were added only once based on WC Medium. **A** RFU over time was measured. Error bars present the standard deviation of all replicates (n=8). **B** Estimated maximum linear growth rates per day (r) of the community. The lake community was most co-limited by both nutrients N and P together due to highest growth rates when both nutrients (N and P) were simultaneously added.

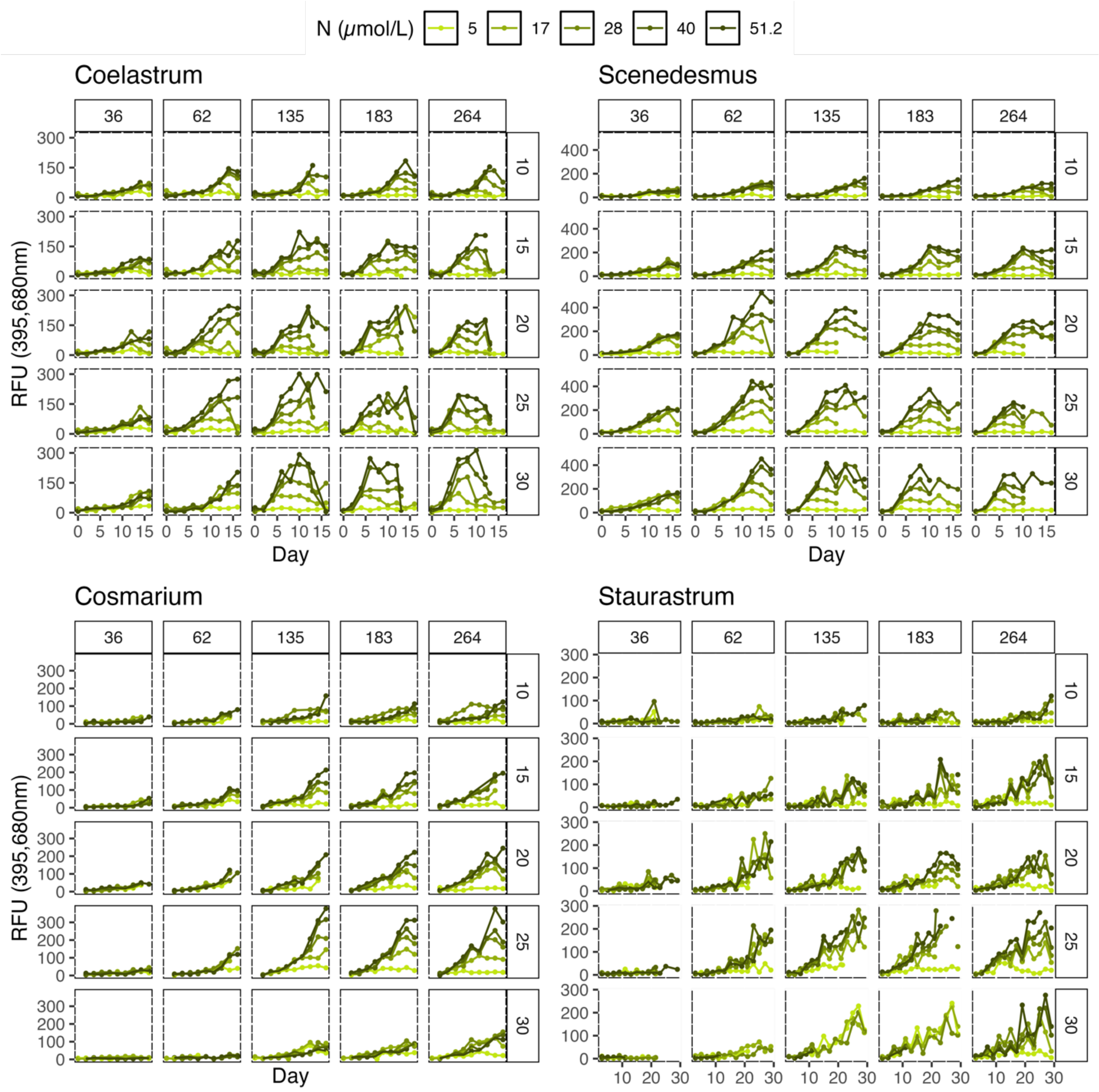

Figure S3 Incubation data (RFU vs time) used for the growth rate calculations, for each light intensity (x-panel grids, μmol photons m^{-2} s^{-1}), temperature (y-panel grid, °C) and nutrient concentration (legend in nitrogen concentration, μmol L^{-1}, see Table 1 for corresponding phosphorus concentrations).

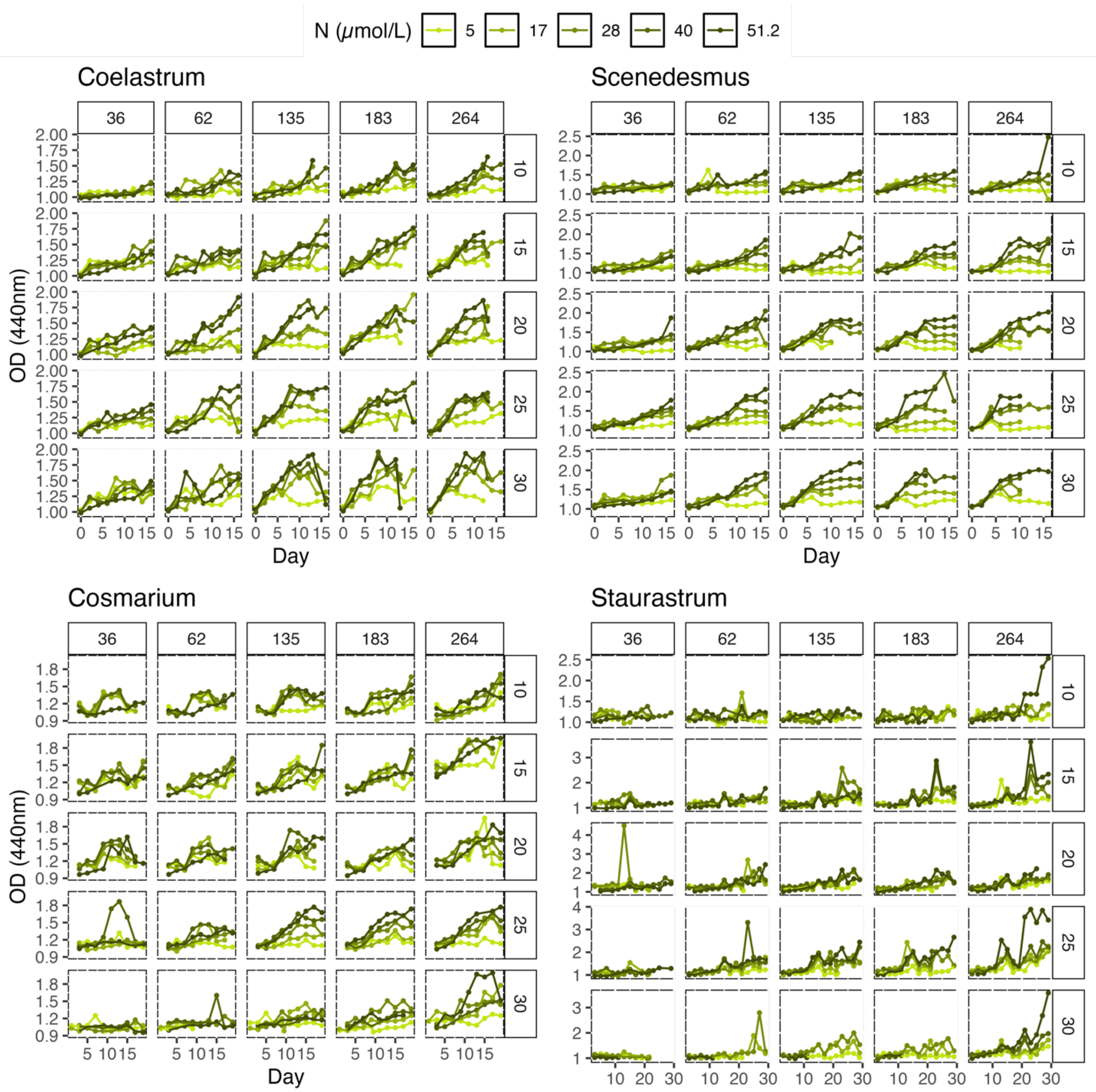

Figure S4 Incubation data (OD vs time) for each light intensity (x-panel grids, μmol photons m^{-2} s^{-1}), temperature (y-panel grid, °C) and nutrient concentration (legend in nitrogen concentration, μmol L^{-1}, see Table 1 for corresponding phosphorus concentrations).

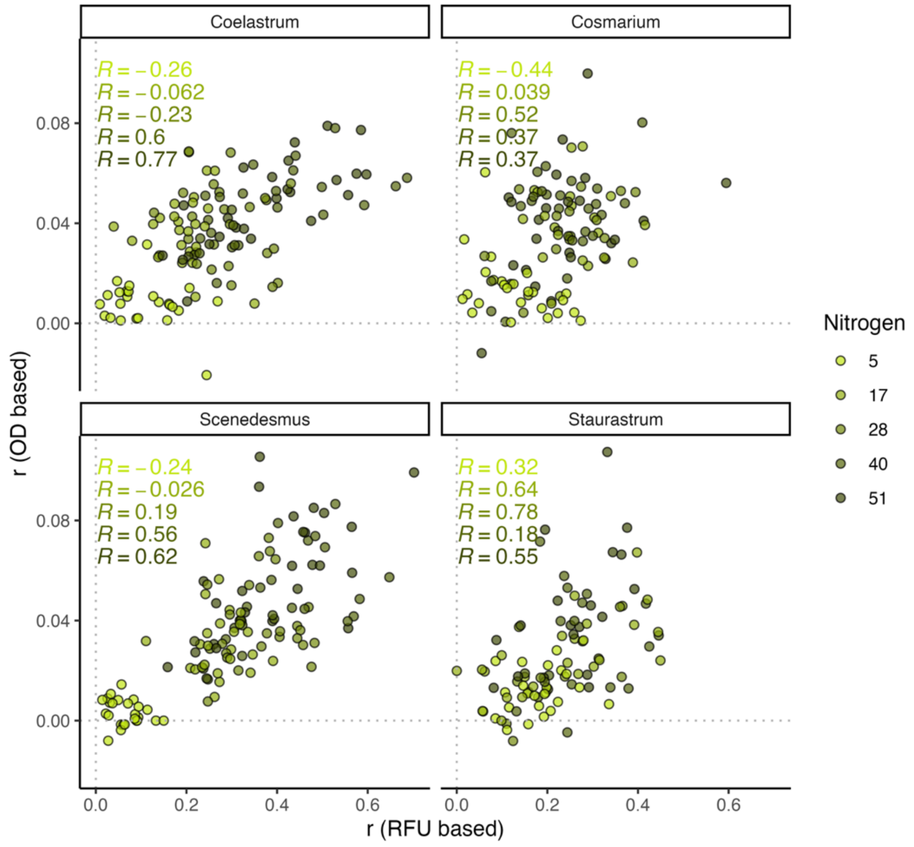

Figure S5 Comparison between growth rates estimated with RFU data (used for the results of this study) and with OD data. Colour of the circles present the different nutrient concentration and *R* the Pearson Coefficient of Correlation for each nutrient level. Although the relationship between these two growth estimates depended on the treatment conditions, the model outputs were comparable (exception was *Staurastrum*, see Appendix S1: Table S1).

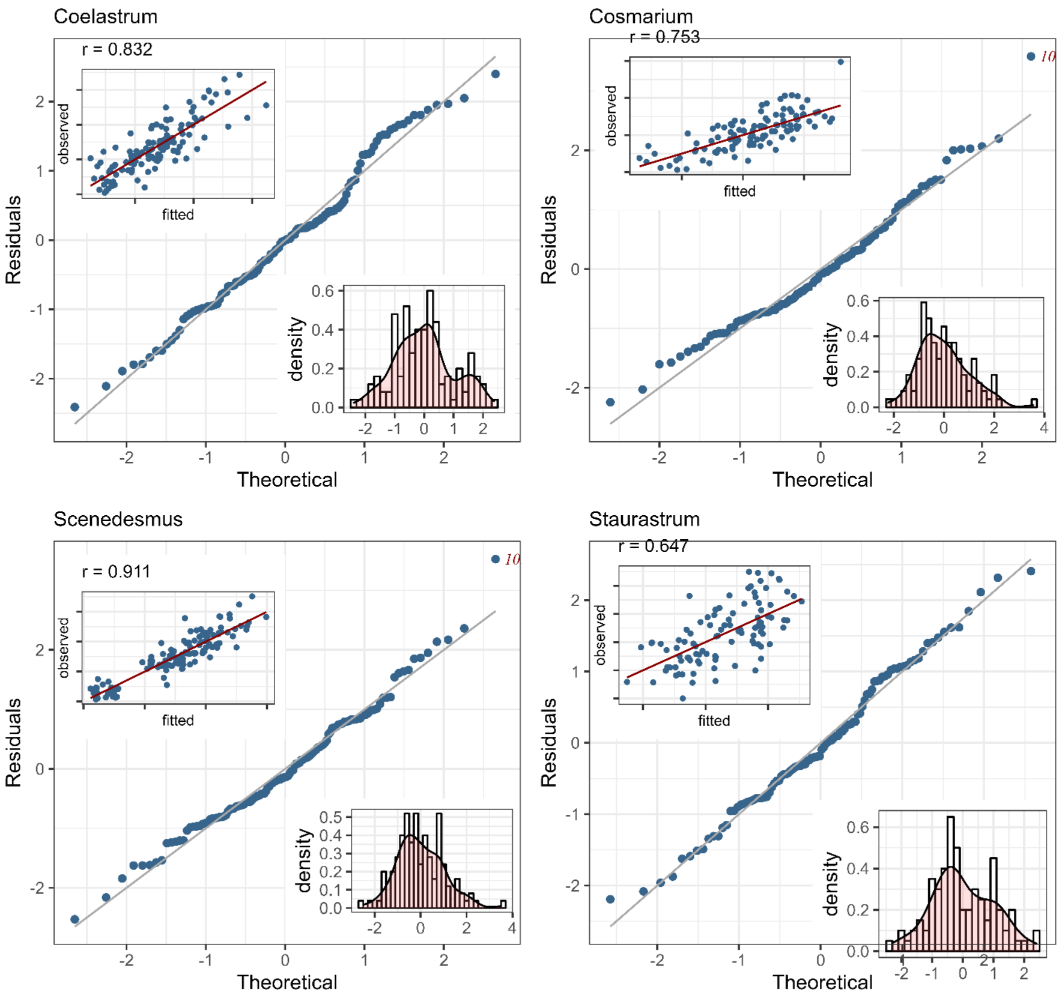

Figure S6 Model validation via R package *gamlss.ggplots.* Quantile-quantile plots of normalized quantile residuals from the temperature-resource interaction model. For *Cosmarium* and *Scenedesmus*, one outlier each (here marked with red letters) was excluded for the models.

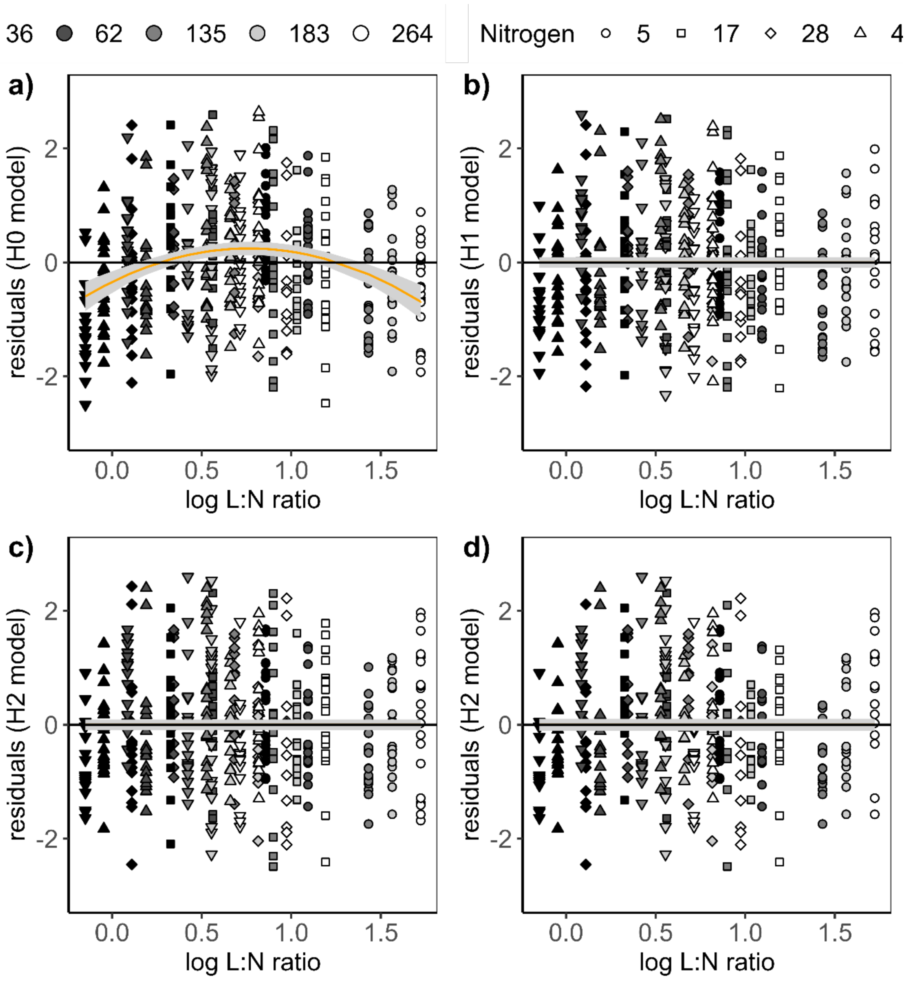

Figure S7 Normalized quantile residuals of the different models (H0=null model, H1=resource interaction model, H2= temperature-resource model) along light:nutrient supply ratios (log-transformed). The temperature-resource model (c,d) shows significantly fewer residual patterns compared to the null model (a). The null model overestimates the growth rates at extreme resource supply when light is low and nutrients high, but the temperature-resource model shows no such deviation from 0. Panel d) shows residuals of H2 model without *Staurastrum*, as *Staurastrum* showed best model predictions with the H0 model.

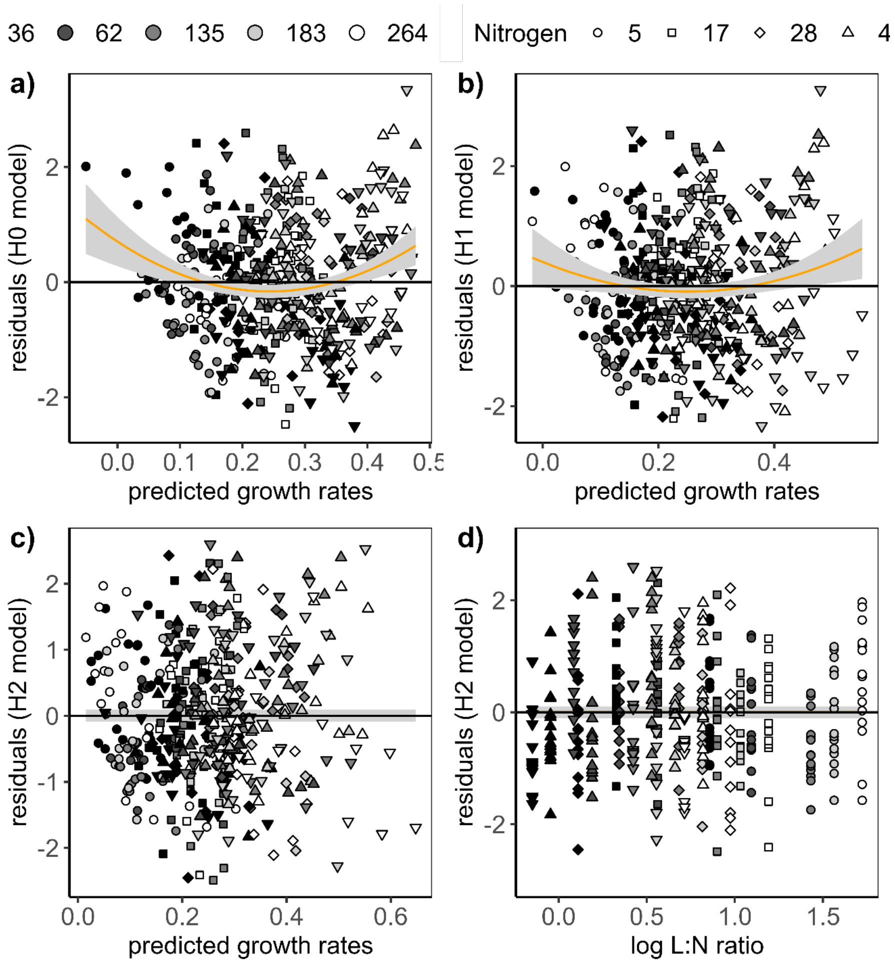

Figure S8 Normalized quantile residuals of the different models (H0=null model, H1=resource interaction model, H2= temperature-resource model) against predicted growth rates. Residuals are standardized as species are visualized together in one plot. The temperature-resource model shows fewer residual patterns than the null model. Panel d) presents residuals of H2 model without *Staurastrum*.

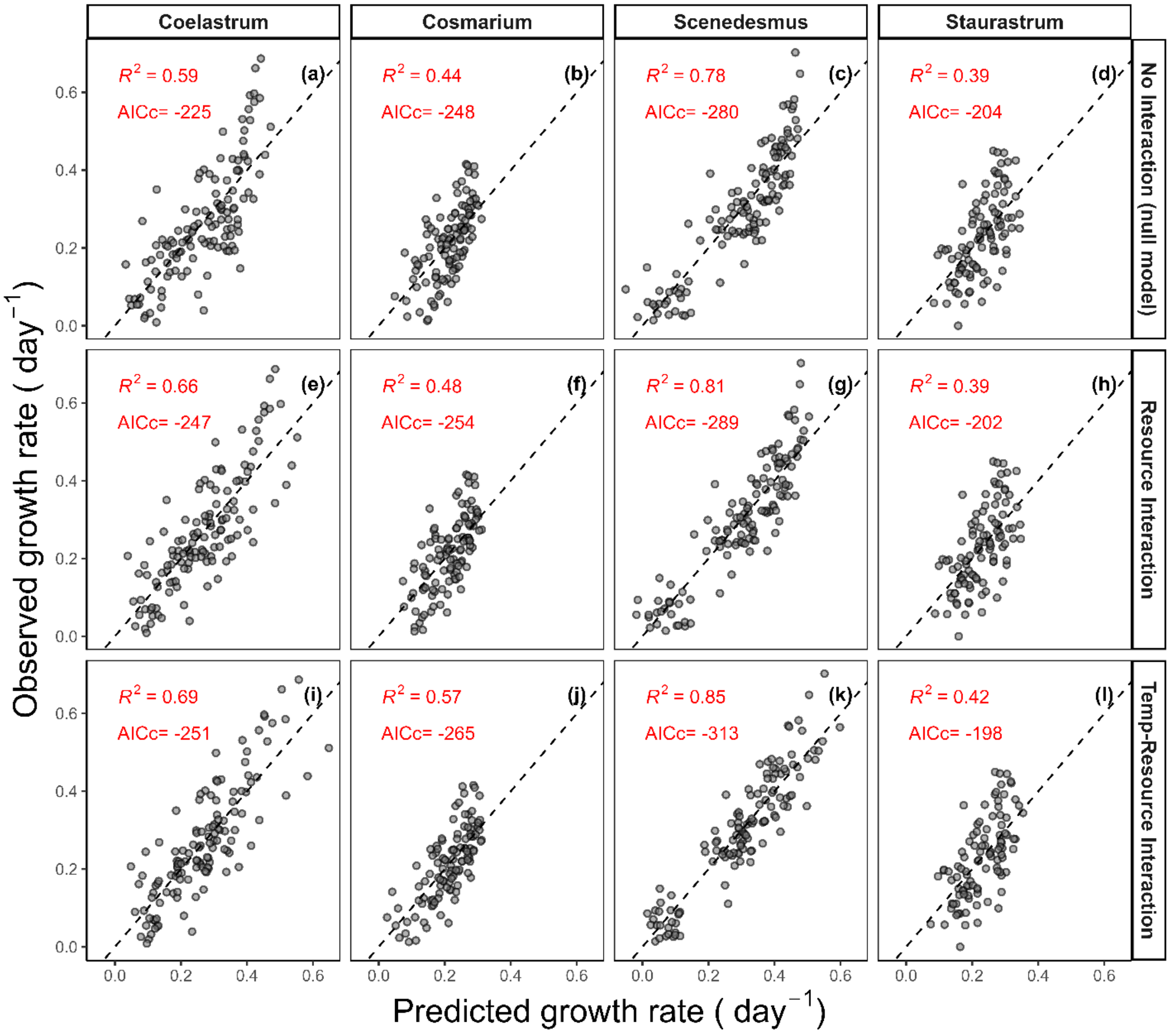

Figure S9 Comparison between the observed growth rates and growth rates predicted by the three models. **a-d** Observed growth rates vs predicted growth rates by the null model (independent effects of temperature, light and nutrients) **e-h** comparison between observed growth rates vs predicted growth rates by the resource interaction model used to test for H1 **i-l** comparison between observed growth rates vs predicted growth rates by the temperature-resource interaction model used to test for H2. Red letters present *Pearson correlation coefficient R* and AICc. Dashed line present 1:1 line.

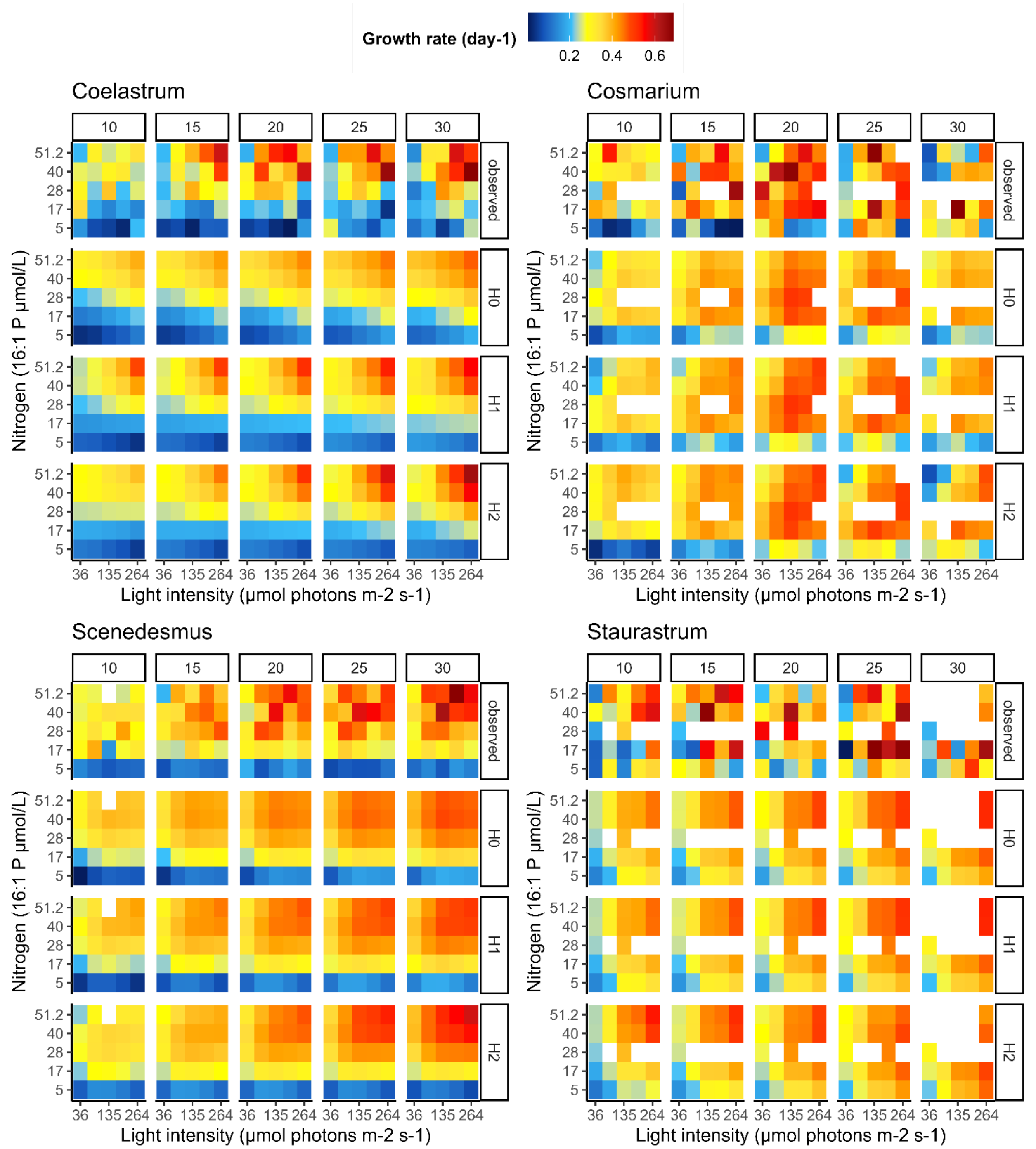

Figure S10 Observed and predicted growth rates along the used nutrient and light gradient across temperatures and species fitted by the different models (H0= null model, H1= resource interaction model, H2= temperature-resource model) White spaces in the Response Surface present missing values due to contaminations.

Table S1 Model outputs with the growth rates based on RFU data and OD data. Given are the effective degrees of freedom (edf), the AIC and AICc values. Except for *Staurastrum*, the H2 model had the best AICC and AIC values (AIC and AICC showed comparable outputs) for both growth rates (based on RFU and OD). Different to the RFU based growth rates, the growth rates of *Staurastrum* based on the OD were, as the other species, best fitted with the H2 model (best AICc and AIC).

		**Outcome based on RFU data**			**Outcome based on OD data**		
	model	edf	AIC	AICc	edf	AIC	AICc
Coelastrum	(H0)	6.4	-227	-226	10.4	-759	-757
	(H1)	7.7	-248	-247	11.5	-776	-773
	(H2)	11	-253	-251	14.6	-789	-785
Cosmarium	(H0)	10.3	-251	-248	7.4	-596	-595
	(H1)	11.5	-257	-254	8.5	-600	-598
	(H2)	14.9	-270	-265	12	-603	-600
Scenedesmus	(H0)	10.1	-282	-279	7.6	-731	-730
	(H1)	11.3	-292	-289	9.0	-755	-754
	(H2)	14.9	-317	-313	13.6	-767	-763
Staurastrum	(H0)	7.6	-206	-204	12.5	-577	-572
	(H1)	8.5	-204	-202	13.6	-587	-583
	(H2)	12.1	-202	-198	16.7	-587	-580
